# PhageCGRNet: Integrating Chaos Game Representation of Genomes with Convolutional Neural Network for accurate phage host classification prediction

**DOI:** 10.1371/journal.pcbi.1014130

**Published:** 2026-04-09

**Authors:** Ting Wang, Zu-Guo Yu, Jinyan Li, Xuan Lin

**Affiliations:** 1 National Center for Applied Mathematics in Hunan & Key Laboratory of Intelligent Computing and Information Processing of Ministry of Education, Xiangtan University, Hunan, China; 2 Hunan Provincial University Key Laboratory for Big Data Analysis and Application, Hunan First Normal University, Changsha, Hunan, China; 3 Faculty of Computer Science and Artificial Intelligence, Shenzhen University of Advanced Technology, Shenzhen Guangdong, China; 4 School of Computer Science, Xiangtan University, Xiangtan, Hunan, China; University of Western Ontario: Western University, CANADA

## Abstract

Phages (or bacteriophages) play a critical role in microbial communities, and accurately predicting the hosts of phages is essential for understanding the dynamics of these viruses and their impact on bacterial populations. In the prediction of classification of phage hosts, feature extraction is a critical step that directly affects the accuracy of the predictions. Among the techniques used for feature extraction, *k*-mers are the most commonly employed method. Although many methods based on *k*-mers have been proposed, these methods typically use only the frequency information of *k*-mers as features. However, when frequencies are identical, the frequency information of these *k*-mers becomes less useful. To address this limitation, we propose a novel method called PhageCGRNet, which not only utilizes the frequency information of *k*-mers but also incorporates the positional information of *k*-mers. In our method, we represent each genome sequence as a three-dimensional matrix containing *k*-mers frequency features and positional features, and then utilize the Convolutional Neural Network model to predict the host category. Specifically, we combine the frequency information of *k*-mers directly extracted from the sequences with the positional information of *k*-mers obtained using the Chaos Game Representation method to construct the feature matrix, which serves as the input to the Convolutional Neural Network. We conducted experiments on two benchmark datasets, and compared PhageCGRNet with existing advanced methods for phage host classification. The experimental results demonstrate that PhageCGRNet achieves higher accuracy at both taxonomy levels of species and genus on these two datasets compared to other state-of-the-art methods.

## Author summary

With the increasing prevalence of drug-resistant bacterial pathogens against major clinically used antibiotics and the slow progress in the antibiotic research and development pipeline, the ability of phages to specifically target and kill bacteria has made them increasingly important in antibacterial treatment. To achieve effective phage therapy, it is essential to accurately predict the interactions between phages and their hosts. However, most existing prediction methods depend solely on *k*-mers frequency information, often overlooking the importance of positional information within sequences. To address this limitation, we propose a novel phage host prediction method in this study. The model outperforms current state-of-the-art methods in host classification performance on two benchmark datasets, providing robust computational tool support for advancing the practical application of phage therapy.

## 1. Introduction

Phages are recognized as the most abundant and possibly the most diversified microorganisms on the planet [1]. Phages usually have specific host, they bind to specialized receptors on the surface of host cells and inject their genetic material into host cells [2]. Since phages are able to attack host bacteria precisely and efficiently without harming humans or other organisms, they can be utilized to selectively kill pathogenic bacteria thereby overcoming the challenges posed by antibiotic resistance [3]. Meanwhile, by changing the DNA sequence of phage, customized phage can be designed for gene delivery, gene therapy or tumor treatment [4]. So it is important to study phages, but traditional laboratory cultivation of phages requires a lot of time and money [5]. Moreover, less than 1% of hosts have been cultivated in the laboratory [6]. Next-generation sequencing technology provides new methods for discovering new phages [7]. The dramatic increase in phage sequencing data makes host identification difficult and limit research on phage host classification. Therefore, it is necessary to develop tools to classify phage hosts more accurately.

In recent years, a variety of computational methods have been proposed to predict phage host classification, which can be broadly divided into alignment approaches and alignment-free approaches. Alignment-based approaches can be implemented through two main strategies. The first strategy involves identifying shared segments between phages and hosts [8]. These approaches include matching CRISPR spacer sequences, identification of related prophages in host genomes, genes transferred horizontally between phage and host. However, there are limitations to using spacer sequences to make phage host predictions, and spacer sequences do not exist for many hosts [9]. The second strategy relies on phage-phage similarity [10]. Instead of searching for direct phage-host matches, these methods infer hosts by comparing the query phage against a database of phages with known hosts, based on sequence similarity. For example, RaFAH [11] predicts hosts by searching phage proteins against a database of protein families using hidden Markov models and applying a random forest classifier to the resulting alignment scores. Similarly, vHULK [12] also relies on protein similarity for host prediction. It compares phage proteins against a prokaryotic virus database. The alignment significance scores are transformed and used as input features. Finally, a multilayer perceptron neural network is employed to predict the host.

Alignment-free methods include abundance profiling methods and nucleotide composition methods [13]. Among these, the nucleotide composition method is based on the observation that phages and their hosts often exhibit similar patterns in codon usage or *k*-mers (*k*-mers denote DNA segments of length *k*). Additionally, phages infecting the same host tend to share similar *k*-mer characteristics due to host-specific selection or shared evolutionary history. Therefore, the similarity in the frequency of *k*-mers has been widely used in phage host prediction. These *k*-mers can serve as feature extraction techniques in host classification algorithms. The extracted features are then input into machine learning algorithms to carry out phage host classification [14–21]. One prominent approach that relies solely on phage sequences is DeepHost [22]. It encodes sequences as 3D matrices and uses the convolutional neural network model to classify hosts of phage. In contrast, several other methods leverage both phage and host data. For instance, CHERRY and PHPGCA establish networks between phages and their hosts. Specifically, CHERRY [23] constructs a knowledge graph between phages and hosts, and then uses the graph convolutional neural network model to predict the species of hosts. PHPGCA [24] also constructs a heterogeneous graph between phages and hosts, and then augments the node representations with the graph contrastive learning algorithm to consider the host prediction task as a recommendation task. Another method, CL4PHI [25] uses Frequency Chaos Game Representation (FCGR) [26] to extract sequence features and then uses the contrastive learning algorithm to identify phages infecting the same host to predict phage host relationships. Differently, PB-LKS [27] utilizes the local *k*-mer similarities of the most similar genomic fragments between phages and hosts as features. It then uses a Random Forest model to predict the interaction between phages and their hosts.

While several existing approaches, such as DeepHost [22] and CL4PHI [25], have demonstrated the utility of combining *k*-mer frequency maps with Convolutional Neural Network (CNN) model [28] for host prediction, they primarily rely on frequency information alone. In contrast, the key innovation of our method, PhageCGRNet, lies in its novel feature representation. We integrate the *k*-mer frequency with the positional information derived from Chaos Game Representation (CGR) into a unified three-dimensional feature matrix. This integration enables our model to simultaneously learn not only what *k*-mers are present but also where they are located within the genomic sequence, thereby capturing a more comprehensive set of sequence features.

In this work, we propose a new approach for phage host classification called PhageCGRNet. In addition to directly extracting features from genome sequences, we also employ the CGR [29] to extract features anew. Specifically, we obtain sequence information by considering the frequency of the *k*-mers of the sequences and the corresponding average position of the *k*-mers in the CGR plots. This integrated information is then input as feature matrices into the CNN model, which learns the characteristics of phages. These learned phage features are subsequently used to predict their corresponding hosts. The combination of frequency and position information of *k*-mers in feature matrices provides more comprehensive representation, which is then integrated with deep learning algorithm CNN for the purpose of more accurately predicting. Notably, our method employs *k*-mer frequency and positional information to characterize phage genomes. Host prediction is achieved by comparing the sequence differences among phages to identify genomic signatures associated with specific hosts, rather than through direct sequence alignment between phages and their hosts.

## 2. Results

### 2.1. Datasets

In this work, we used two datasets that were previously employed by DeepHost [22] and CHERRY [23], which we respectively refer to as Dataset 1 and Dataset 2. Dataset 1 initially consists of 8,756 phages. These phages are filtered following the data selection approach described in DeepHost [22]. After the filtering process, we retain 8,595 phage sequences belonging in 49 family-level and 72 genus-level host taxonomies, and 7,483 phage sequences belonging in 118 species-level host taxonomies in Dataset 1. Dataset 2 comprises 1,875 phages, covering 206 species-level,108 genus-level and 67 family-level host taxonomies. For both Dataset 1 and Dataset 2, we randomly partition them at a ratio of 8:1:1 to obtain the training set, validation set and test set.

### 2.2. Performance evaluation metircs

In order to compare our model with state-of-the-art methods, we use accuracy, precision, recall, F1-score and top-*k* accuracy to measure the performance of the model. The formula for this accuracy metric is presented in Equation (1):


$$Accuracy=\;\frac{Number\;of\;predictions\;correct}{Total\;number\;of\;predictions}.$$ (1)

Host taxonomy prediction is a multi-class prediction problem. Therefore, we use macro precision, macro recall, and macro F1-score for performance evaluation. The calculation formulas are as follows:


$$\text{Macro Precision }=\;\frac{\text{1}}{n}\sum_{i=\text{1}}^{n}{Precision}_{i},$$ (2)

where, ${Precision}_{i}=\frac{{TP}_{i}}{{TP}_{i}+{FP}_{i}},\;n\text{ is the number of classes}.$ ${TP}_{i}\;$ is the number of samples that actually belong to class *i* and are correctly predicted as class *i*. ${FP}_{i}$ is the number of samples that do not actually belong to class *i* but are incorrectly predicted as class *i.*


$$\text{Macro Recall }=\;\frac{\text{1}}{n}\sum_{i=\text{1}}^{n}{Recall}_{i},$$ (3)

where, ${Recall}_{i}=\frac{{TP}_{i}}{{TP}_{i}+{FN}_{i}}$. ${FN}_{i}$ is the number of samples that actually belong to class *i* but are incorrectly predicted as another class.


$$\text{ Macro F1}-\text{score }=\frac{\text{1}}{n}\sum_{i=\text{1}}^{n}{F\text{1}-score}_{i},$$ (4)

where, ${F\text{1}-score}_{i}=\text{2}\times \frac{{Precision}_{i}\times {Recall}_{i}}{{Precision}_{i}+{Recall}_{i}}.$

### 2.3. Comparison of performance

#### 2.3.1. Baseline methods.

We conducted experiments on two benchmark datasets: Dataset 1 (Cherry) and Dataset 2 (DeepHost). To comprehensively evaluate the performance of our methods, we compared it with several state-of-the-art approaches. These include RaFAH [11] and vHULK [12], DeepHost [22], CHERRY [23], PHPGCA [24], CL4PHI [25], and PB-LKS [27]. For the RaFAH [11], vHULK [12], PHPGCA [23] and PB-LKS [26], we reproduced the experiments using the parameter settings provided in the original paper on Dataset 1. For the other three methods (DeepHost [22], CHERRY [23], and CL4PHI [25]), we used the results reported in the CL4PHI [25] on Dataset 1. For Dataset 2, due to changes in the dataset division, we re-implemented all seven methods using the parameter settings specified in their respective papers.

#### 2.3.2. Experimental setup.

In our experiments, the length of the *k*-mers used in the feature extraction stage is set to 7. This resulted in a feature matrix with a size of 3 × 128 × 128. Our CNN model consists of two convolutional layers. Each convolutional layer has a kernel size of 5 × 5, a stride of 1, and padding of 2. After each convolutional layer, there is a pooling layer with a kernel size of 2 × 2 and a stride of 2. Additionally, the fully connected layer contains 512 neurons. We provided the detailed parameter settings in Table 1. Meanwhile, we compared our proposed PhageCGRNet with other advanced methods, including RaFAH [11], vHULK [12], DeepHost [22], CHERRY [23], PHPGCA [24], CL4PHI [25], PB-LKS [27]. We comprehensively evaluated the performance of our method across two datasets at three taxonomic levels (species, genus, and family) using four metrics: accuracy, precision, recall, and F1 score. The detailed results are presented in Table 2. Bold values indicate the best performance for each metric. Since RaFAH [11] makes predictions at genus level, it is represented by “-” at species level. As shown in Table 2, on Dataset 1, our method outperformed all other state-of-the-art methods across all metrics at both the genus and family levels. At the species level, although precision was slightly lower than that of CL4PHI, our method achieved superior results in all other metrics. On Dataset 2 (Table 3), our method achieves an accuracy of 79.68% at the species level, surpassing the second-best method, PHPGCA, by 6.42%; achieves 89.30% accuracy at the genus level, exceeding PHPGCA by 8.55%; and attains 91.44% accuracy at the family level, an improvement of 4.76%. Furthermore, our method consistently outperforms all other methods in the remaining evaluation metrics.

**Table 1:** Detailed configuration of the CNN architecture.

Layer	Parameter Configuration	Input Dimension	Output Dimension
Conv1	Conv2d(3, 64, kernel_size = 5, stride = 1, padding = 2), RuLu	(3,128,128)	(64,128,128)
Pool1	MaxPool2d(kernel_size = 2, stride = 2)	(64,128,128)	(64,64,64)
Conv2	Conv2d(64, 128, kernel_size = 5, stride = 1, padding = 2),RuLu	(64,64,64)	(128,64,64)
Pool2	MaxPool2d(kernel_size = 2, stride = 2)	(128,64,64)	(128,32,32)

**Table 2:** Comprehensive Evaluation of host prediction performance on Datasets1 and comparison with other methods.

Methods	Species				Genus				Family			
	Accuracy	Precision	Recall	F1-score	Accuracy	Precision	Recall	F1-score	Accuracy	Precision	Recall	F1-score
RaFAH [11]	–	–	–	–	0.9427	0.8684	0.8245	0.8320	0.9656	0.9128	0.9176	0.9066
vHULK [12]	0.9122	0.7627	0.7483	0.7424	0.9302	0.8209	0.8318	0.8172	0.9636	0.9067	0.8879	0.8841
DeepHost [22]	0.9105	0.6707	0.6749	0.6611	0.9479	0.8364	0.8350	0.8274	0.9561	0.9229	0.9111	0.9097
CHERRY [23]	0.7597	0.6871	0.6638	0.6577	0.8264	0.7596	0.7275	0.7292	0.8993	0.8489	0.8171	0.8171
PHPGCA [24]	0.9240	0.7567	0.7068	0.7154	0.9488	0.8457	0.7797	0.7976	0.9639	0.9291	0.927	0.9192
CL4PHI [25]	0.9279	0.7791	0.7547	0.7576	0.9613	0.8663	0.8259	0.8356	0.9686	0.9501	0.9141	0.9115
PB-LKS [27]	0.9167	0.7719	0.7464	0.7560	0.9486	0.8333	0.8197	0.8254	0.9662	0.8824	0.8784	0.8803
PhageCGRNet	**0.9412**	0.7754	**0.7793**	**0.7676**	**0.9651**	**0.8985**	**0.8365**	**0.8536**	**0.9790**	**0.9608**	**0.9468**	**0.9500**
Improvement (%)	1.33	–	2.46	1.00	0.38	3.22	0.15	1.80	1.04	1.07	3.27	3.08

**Table 3:** Comprehensive Evaluation of host prediction performance on Datasets2 and comparison with other methods.

Methods	Species				Genus				Family			
	Accuracy	Precision	Recall	F1-score	Accuracy	Precision	Recall	F1-score	Accuracy	Precision	Recall	F1-score
RaFAH [11]	–	–	–	–	0.8127	0.6086	0.5936	0.5913	0.8678	0.7671	0.7599	0.7517
vHULK [12]	0.7006	0.2405	0.4657	0.4514	0.7767	0.5805	0.5757	0.5614	0.8369	0.7361	0.7356	0.7233
DeepHost [22]	0.6846	0.3850	0.4187	0.3892	0.7881	0.5346	0.5322	0.5168	0.8351	0.7369	0.6980	0.6857
CHERRY [23]	0.7153	0.5231	0.554	0.5208	0.7990	0.6354	0.6669	0.6444	0.8668	0.7163	0.7153	0.7100
PHPGCA [24]	0.7317	0.5286	0.5136	0.5137	0.8009	0.6615	0.6517	0.6481	0.8725	0.7596	0.7275	0.7292
CL4PHI [25]	0.7326	0.5769	0.5638	0.5612	0.8075	0.6822	0.6733	0.6685	0.8603	0.7361	0.7356	0.7233
PB-LKS [27]	0.7220	0.5069	0.4668	0.4741	0.7994	0.679	0.6788	0.6667	0.8527	0.7203	0.7021	0.7180
PhageCGRNet	**0.7968**	**0.6604**	**0.681**	**0.6618**	**0.8930**	**0.7663**	**0.7532**	**0.7443**	**0.9144**	**0.8067**	**0.7879**	**0.7841**
Improvement (%)	6.42	8.35	11.72	10.06	8.55	8.41	7.44	7.58	4.76	4.71	5.23	5.49

**Fig 1:**
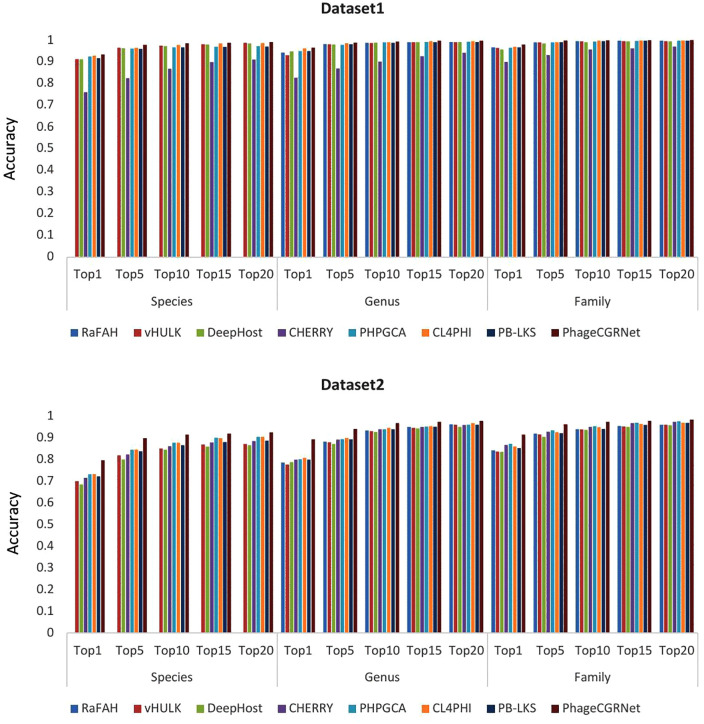
Accuracy at Top k for Dataset 1 and Dataset 2 at the species, genus, and family levels.

**Fig 2:**
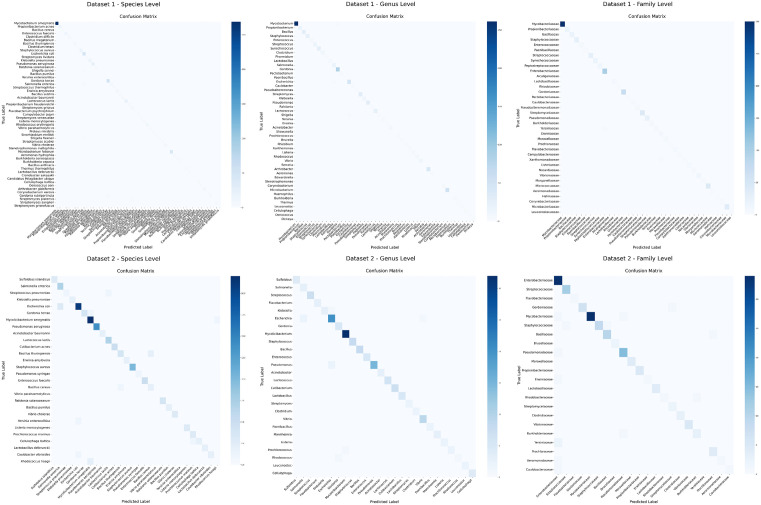
Confusion matrices illustrating the classification performance of PhageCGRNet.

In addition to the above evaluation metrics, we also employ Top-*k* accuracy (*k* = 1,5,10,15,20) to assess prediction performance. The bar chart in Fig 1 demonstrates that our method achieves high performance across all *k* values. Furthermore, the confusion matrix presented in Fig 2 provides a detailed visualization of the model’s classification behavior across different host categories. The diagonal elements represent the number of correct predictions for each class, while the non-diagonal elements reveal the distribution of misclassifications between categories. The diagonal elements are markedly darker, indicating that the majority of predictions fall on the correct class. Non-diagonal elements are sparse and light, confirming a low rate of misclassification.

#### 2.3.3. Statistical test.

To further illustrate that our method is more accurate than the CL4PHI method on Dataset 1, we assess the statistical significance of the difference between the values of the evaluation metrics of PhageCGRNet and CL4PHI. First, we repeat the experiment 30 times respectively. We test normality with the Kolmogorov-Smirnov test at the 0.05 P-value [30]. The results indicate that the data do not exhibit a normal distribution. Finally, we adopt the Wilcoxon signed-rank test [31]. The accuracy of PhageCGRNet is significantly higher than CL4PHI (P-value < 0.05) and a large effect size (Cliff’s Delta [32] = 0.4775).

### 2.4. Model performance under varying similarity between training and test sets

**Fig 3:**
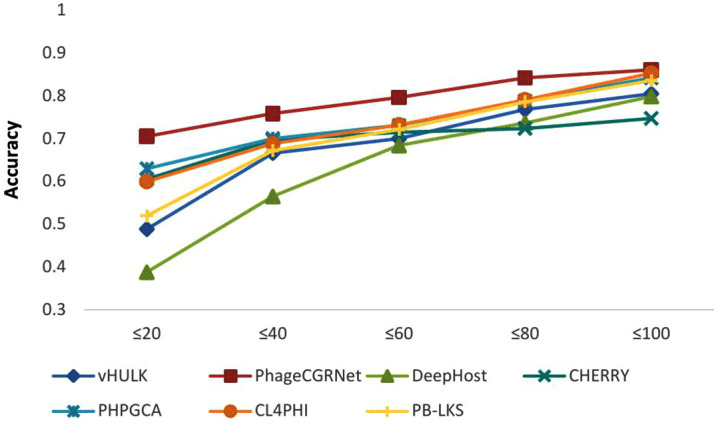
Species-level host prediction performance is influenced by the similarity between training and test sets.

We conducted a stratified similarity analysis using the Dashing tool to compute the similarity between test set and the training set. We divided the test set according to the similarity (≤20%, ≤ 40%, ≤ 60%, ≤ 80%, and ≤100%). The prediction accuracy of all competing models was then evaluated within each bin at the species taxonomic level of Dataset 2, with the results presented in Fig 3. The analysis reveals that while the performance of all models improves as the similarity between test samples and the training set increases, our method achieves the highest accuracy in every single similarity bin. This confirms that the advantage of our approach is robust and does not depend critically on a specific similarity distribution between the training and test sets.

### 2.5. Ablation experiment

In order to ascertain the factors that influence the accuracy of the results obtained, we conducted ablation experiments. As shown in Table 4. The experimental setup was designed as follows: First, we employed *k*-mer frequency features and CGR positional features as independent inputs for classification prediction using Multi-Layer Perceptrons (MLP) model [33]. Subsequently, these two features were integrated into a composite 3D feature matrix for MLP-based classification. Meanwhile, we repeated the same workflow with a convolutional neural network (CNN), performing classification tasks using each individual feature and their combined form (frequency and positional features). The results demonstrated that: for both CNN and MLP models, the CGR-derived positional features led to a greater improvement in accuracy (as detailed in Table 4) compared to *k*-mer frequency features. Moreover, the synergistic use of frequency and positional information played an indispensable role in further optimizing prediction accuracy.

**Table 4:** Ablation experiments in Dataset 1 and Dataset 2.

Feature	Datset 1			Dataset 2		
	Species	Genus	Family	Species	Genus	Family
w/ f	0.8987	0.9188	0.9396	0.7001	0.7166	0.8128
w/ p	0.9032	0.9298	0.9446	0.7497	0.7614	0.8312
w/o p	0.9146	0.9453	0.9511	0.7433	0.7540	0.8289
w/o f	0.9252	0.9464	0.9558	0.7701	0.8021	0.8503
w/o CNN	0.9186	0.9489	0.9614	0.7505	0.8075	0.8617
PhageCGRNet	**0.9412**	**0.9651**	**0.9790**	**0.7968**	**0.8930**	**0.9144**

Note: w/f indicates using only *k*-mer frequency features with the MLP model, w/p indicates using only *k*-mer position features with the MLP model, w/o p indicates using *k*-mer frequency features but excluding position features with the CNN model, w/o f indicates using *k*-mer position features but excluding frequency features with the CNN model, and w/o CNN indicates using both *k*-mer frequency and position features but employing the MLP instead of the CNN for prediction.

In order to explore whether CNN contributes to enhance accuracy of our method, we conducted a comparative analysis of model performance before and after CNN integration (using an MLP model as the baseline). The experimental framework comprised two dimensions: (i) Single-feature comparison: Independent evaluations using *k*-mer frequency feature matrices and CGR positional feature matrices to assess classification performance differences between CNN and MLP under single-feature conditions; (ii) Composite-feature validation: Fusion of both feature types into a 3D matrix to validate model behaviors with hybrid inputs. Experimental results indicated: in single-feature scenarios, CNN outperforms the MLP baseline in species-level/genus-level/family-level classification tasks across two datasets (specific values in Table 4). When using combined features, the CNN model exhibits stronger capabilities in capturing feature interactions, leading to improved accuracy on both two datasets. These results conclusively demonstrate that CNN integration effectively strengthens feature representation learning, thereby improving classification accuracy.

### 2.6. Parameter analysis

**Fig 4:**
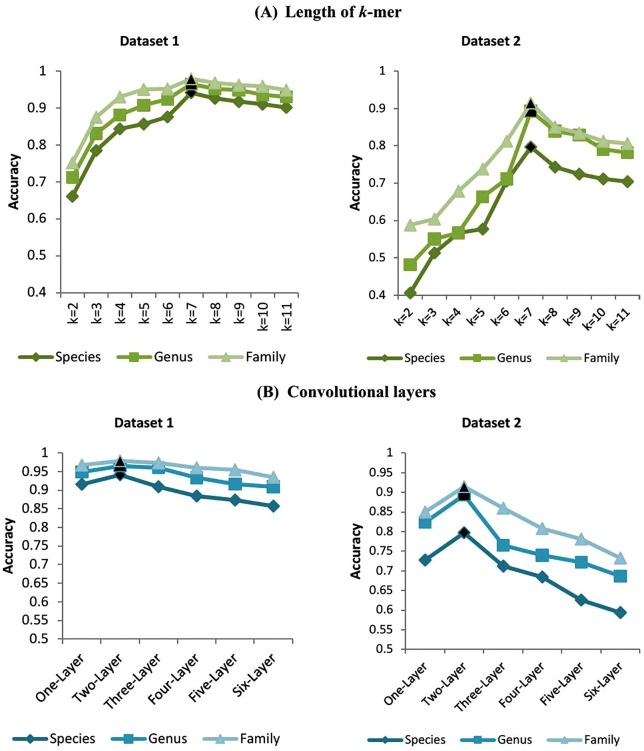
Host prediction accuracy under different parameter settings. **(A)** Host prediction accuracy of PhageCGRNet on Datasets 1 and 2 for different values of ***k.* (B)** Host prediction performance using convolutional layers with varying depths on two datasets. In both panels, the optimum values are indicated in black.

Since our method is based on *k*-mers, the value of *k* is a crucial parameter. To determine its optimum value, we carried out an experimental study. We tested our method using a range of *k*-mer lengths. For each *k*-mer length, we evaluated the resulting accuracy. The results of these experiments are shown in Fig 4(A). From this figure, we can clearly observe the relationship between the value of *k* and the prediction accuracy. Initially, as the value of *k* increases, the prediction accuracy of the phages-hosts classification also rises. The accuracy reaches its peak when *k* is equal to 7. However, when *k* continues to increase beyond 7, the accuracy starts to decline. Based on these findings, we conclude that the optimal value of *k* for our method is 7.

Additionally, we also investigated the impact of the number of convolutional layers on the model’s performance. To achieve this, we conducted experiments with different numbers of convolutional layers to determine the optimal number. As shown in Fig 4(B), the experimental results indicate that using two convolutional layers yields the best performance. This may be attributed to the fact that two convolutional layers strike a good balance between model complexity and computational efficiency, allowing for the capture of sufficient feature information without leading to over fitting or excessive computational resource consumption.

Moreover, we tested four different nucleotide-to-CGR vertex mapping schemes to determine the optimal allocation strategy. Since nucleotides can be assigned to CGR vertices in multiple ways, we specifically examined the impact of assigning the (1, 1) point to different nucleotides. As shown in Table 5, the experimental results indicate that varying assignment schemes had little effect on model accuracy. Therefore, we adopted the mapping: A (0, 0), C (0, 1), G (1, 0), and T (1, 1).

**Table 5:** Comparison of host prediction accuracy using different nucleotide assignment schemes.

Assignment	Dataset 1			Dataset 2		
	Species	Genus	Family	Species	Genus	Family
ACTG	0.9412	0.9651	0.9790	0.7968	0.8930	0.9144
ACGT	0.9386	0.9651	0.9744	0.7807	0.8910	0.9091
ATCG	0.9388	0.9580	0.9756	0.7914	0.8975	0.9037
TCAG	0.9346	0.9627	0.9767	0.7896	0.8938	0.9091

Note: Starting from the origin point (0, 0), the nucleotides are arranged in a clockwise order.

Although there are slight differences in model accuracy among different allocation methods, the disparities are extremely limited. To reasonably explain this phenomenon, we conducted a series of experiments: each allocation method was independently repeated 20 times, with the same random seed used for each experiment to control variables. Subsequently, we performed statistical significance analysis using paired t-tests. As shown in Table 6, we found that the P-values of all allocation methods are significantly greater than the significance threshold of 0.05, indicating that the differences in model performance among different allocation methods do not reach statistical significance. In this work, we adopted the ACTG allocation scheme as the default configuration in subsequent experiments.

**Table 6:** P-values from paired t-tests using different nucleotide assignment schemes.

Assignment	Species				Genus				Family			
	ACTG	ACGT	ATCG	TCAG		ACTG	ACGT	ATCG	TCAG		ACTG	ACGT	ATCG	TCAG
ACTG	\					\					\			
ACGT	0.703	\				0.716	\				0.709	\		
ATCG	0.722	0.715	\			0.741	0.701	\			0.733	0.716	\	
TCAG	0.753	0.706	0.699	\		0.757	0.714	0.729	\		0.712	0.711	0.706	\

### 2.7. Comparison between CNN with other Machine Learning models

**Fig 5:**
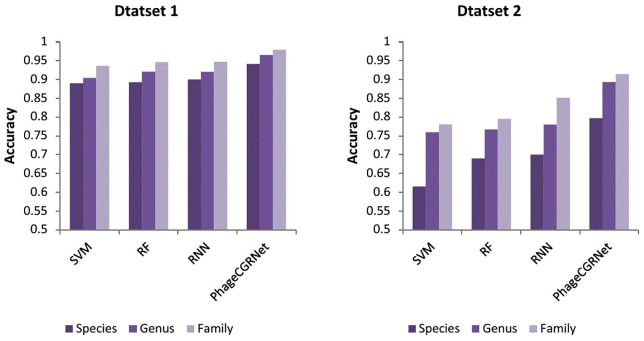
Performance comparison between PhageCGRNet and other machine learning classifiers for family-level, genus-level and species-level phage host classification on Dataset 1 and Dataset 2.

We compared the CNN model of PhageCGRNet with various other machine learning classifiers, covering the Support Vector Machine (SVM) [34] and Random Forest (RF) [35] in the field of traditional machine learning, as well as the Recurrent Neural Network (RNN) [36] in the realm of deep learning. We used our feature extraction method to obtain the input matrices for all classifiers. Fig 5 shows the comparison of the prediction accuracies of each model. To further quantitatively assess the performance differences between the CNN model and other classifiers, we conducted statistical significance tests and effect size analysis. Based on the results of 30 repeated experiments, we employed the Wilcoxon signed-rank test to evaluate statistical significance and calculated Cliff’s Delta effect size (δ) to quantify the magnitude of differences. The test results are summarized in Table 7. From this table, it can be observed that at the species-level, genus-level, and family-level, the P-values are all below 0.05, and multiple Cliff’s Delta effect sizes reach or approach 1. This indicates that the accuracy of the CNN model is significantly higher than that of other classifiers, with large effect sizes. It is clearly visible from Fig 5 and Table 7 that the accuracy of the CNN model is higher than that of other classifiers.

**Table 7:** Statistical Comparison of CNN Models with Other Classifiers.

Comparison Pair	Species		Genus		Family	
	p	δ	p	δ	p	δ
SVM vs CNN	0.000001	1.0000	0.000001	1.0000	0.000001	1.0000
RF VS CNN	0.000001	1.0000	0.000011	0.9229	0.000001	1.0000
RNN VS CNN	0.000018	0.8141	0.000005	0.7937	0.000041	0.8413

### 2.8. Model interpretability and feature analysis

To evaluate the improvement in phage host clustering resulting from the incorporation of CGR positional information, we conducted a quantitative clustering assessment of two representation methods: one relying exclusively on *k*-mer frequency features, and another combines *k*-mer frequency and CGR positional features. Specifically, we calculated their Silhouette Coefficient [37] and Calinski-Harabasz Index (CH) [38]. The results show that the fused CGR and *k*-mer feature set achieves a Silhouette Coefficient of 0.2889 and a CH index of 126.55, which are significantly higher than the values of 0.1216 and 59.44 obtained with *k*-mer features alone. This indicates that phages infecting the same host form tighter and more separated clusters in the high-dimensional feature space, quantitatively validating the enhanced clustering effect after integrating CGR positional information. We effectively overcome the limitations of single features by combining the global distribution of CGR with the local frequency of *k*-mers. Notably, it addresses the issue that *k*-mers alone cannot represent the spatial distribution of repetitive elements, validating the superiority of fusing these two types of information.

**Fig 6:**
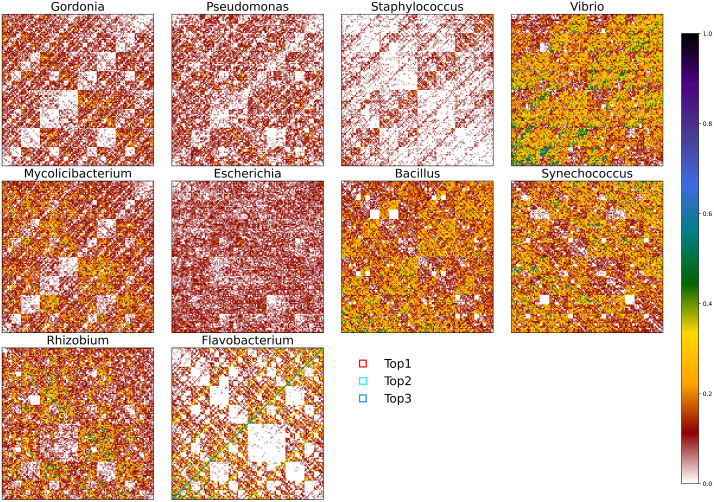
The FCGR plots generated from one sequence each taken from the ten most abundant genus of phages in Dataset 2. The red, cyan, and blue boxes highlight the regions with the highest, second highest, and third highest contributions, respectively.

Jeffrey [28] and Deschavanne et al. [39] noted that CGR can reveal global sequence patterns specific to genomes, including double-spoon structures, diagonal distributions, absence of diagonals, horizontal variation in intensities from top to bottom or reverse, empty patches, and word-rich regions of different shapes. For example, in Fig 6, the blank areas in the AT diagonal regions of Gordonia, Pseudomonas, and Mycolicibacterium stem from the absence of consecutive AA/TT base pairs (or *k*-mers ending with them) and fixed combinatorial AT/TA dinucleotides (or *k*-mers ending with them). The high-density coloring of the AT diagonal in Staphylococcus and Flavobacterium reflects the presence of periodically repeated AT/TA dinucleotides in the sequence. The reduced point density in the CG subquadrant of Flavobacterium indicates the absence of sub-sequences ending with CG. These patterns reveal the global base composition preferences and distribution characteristics of sequences.

To further decipher the sequence patterns recognized by the PhageCGRNet model, we employed game theory-based SHapley Additive exPlanations (SHAP) value [40] to decompose the feature contributions to the model’s prediction outcomes. SHAP values not only quantify the marginal impact of each input feature on individual predictions but also ensure global interpretability consistency. Specifically, we selected genomic sequences from the ten most abundant phage genus in Dataset 2, computed SHAP values for all input-layer features, ranked these features by their contribution scores, and extracted the top three highest-contributing features for each genus. Subsequently, we mapped these features to their corresponding *k*-mer regions (Fig 6), with detailed coordinates provided in Table 8. Our analysis reveals that the regions with the greatest predictive contributions varied across phage categories, with a pronounced clustering in the upper-right quadrant of the CGR plots. Notably, the highest-contributing positions within some genus are not associated with high *k*-mer abundance but rather with relatively low-abundance regions, as exemplified by Staphylococcus, Pseudomonas. This phenomenon arises because low-frequency regions exhibit inter-generic divergence that is statistically more discriminative, whereas high-frequency regions are conserved across multiple genus and thus offer limited taxonomic resolution. These findings underscore that PhageCGRNet effectively captures low-frequency yet high-specificity sequence signatures through its integration of *k*-mer frequency and CGR positional information, thereby providing enhanced discriminatory power for phage classification. To verify that the identified low-frequency, high-specificity *k*-mers are robust to the composition of the phage genome database, we performed repeated subsampling experiments (*n* = 20) and found that the top-contributing *k*-mers remained consistent across subsets (overlap > 85%), confirming that the detected signatures are not substantially biased by the specific dataset used.

**Table 8:** Top three highest-contributing k-mer regions for each phage genus based on SHAP values analysis.

Genus	Top1	Top2	Top3
Gordonia	TACGATT	AAGGTTT	TACGTTT
Pseudomonas	TATGTTT	GTTGTTT	TAGGATT
Staphylococcus	GGCTCCT	CGCTCCT	GCTTCCT
Vibrio	CGCTCCT	CCCTCCT	GGTCCCT
Mycolicibacterium	TAGGTTT	TACGTTT	TACGATT
Escherichia	GGCCCCT	CCCTCCT	GTCTCCT
Bacillus	GGTCCCT	GCCTGCT	ATCCTGG
Synechococcus	GGCCCCT	AGCTCCT	GCCTCCT
Rhizobium	GGGTCCT	CCGTCCT	GCTACCT
Flavobacterium	TACCTTT	ACTGCTT	ACGCACT

Then, we further analyzed the high-contribution sequence patterns identified by SHAP. Specifically, we examined the distribution of the most contributive sequence patterns in phage genomes. Our findings revealed that these patterns are enriched in functional genes critical to phage-host interactions, including tail fiber protein genes (mediating host receptor recognition), phage lysozyme genes (involved in cell wall degradation), tail tape measure protein genes, and transposase genes (regulating genome integration), all of which are well-documented in prior studies [41–44]. PhageCGRNet captures global and local sequence patterns via CGR while integrating *k*-mer frequency data, a dual representation that fuses multi-dimensional information to identify complex patterns.

**Fig 7:**
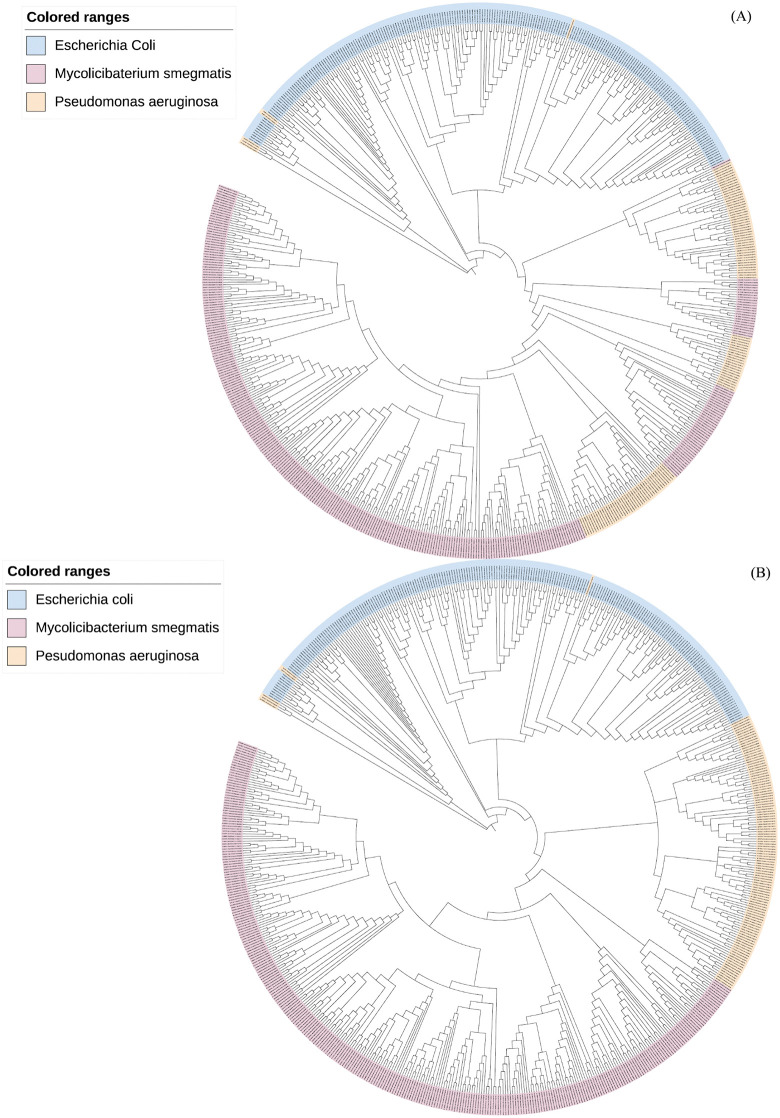
Phylogenetic trees of species. (A) shows the phylogenetic tree constructed using *k*-mer frequency as the feature. (B) shows the phylogenetic tree constructed using both *k*-mer frequency and positional information as features.

Additionally, we selected phages from Dataset 2 that have more than 100 sequences at the species level: 117 sequences from Pseudomonas aeruginosa, 226 sequences from Escherichia coli, and 312 sequences from Mycolicibacterium smegmatis, totaling 655 sequences. Based on the distance calculation in Section 2.4, we calculated the distance matrix of these 665 sequences, and we constructed phylogenetic trees using the Neighbor-Joining [45] method in Mega X, with one tree based solely on *k*-mer frequency (Fig 7(A)) and the other incorporating both frequency and positional information (Fig 7(B)). In comparing Fig 7(A) and 7(B), discrepancies in sequence classification can be observed. Both figures incorrectly classify six Pseudomonas aeruginosa sequences under Escherichia coli. Moreover, Fig 7(A) shows several sequences of Pseudomonas aeruginosa intertwined with those of Mycolicibacterium smegmatis. However, this issue is not present in Fig 7(B), indicating an improved accuracy when utilizing both frequency and positional information as features.

For the six misclassified sequences, we conducted a more detailed analysis. M11912 and M19377 are filamentous phages with a genome length of 5,833 bp, which is significantly shorter than the average genome size of 64,868 bp for the tailed phages that constitute the rest of the Pseudomonas aeruginosa phages in this dataset. Such size differences likely perturbed *k*-mer distribution patterns and impaired feature extraction from CGR positions. And two of the top three contributing regions are the same which makes the feature extraction ineffective and leads to taxonomic misclassification. In addition, sequence NC_031274 erroneously forms a cluster with sequences JX415536, KF981730, EU330206, NC_027395, JX867715 and NC_028903 of Escherichia coli. According to Equation (13) in Section 4.4, we calculated that the average similarity between sequence NC_031274 and the Escherichia coli sequence group is 0.5323. However, the average cosine similarity between NC_031274 and other Pseudomonas aeruginosa sequences is below 0.5, which led to the classification error. Furthermore, three sequences, HG962376, JQ307387 and NC_028879, formed independent subgroups. We computed the pairwise similarity between each of these sequences and other Pseudomonas aeruginosa sequences, and found that all values were below 0.3.

### 2.9. Case study

We selected three phages—vB_PaeP_4029, vB_PaeP_4032, and vB_PaeP_4034—from the clinical case reported by Ferry et al. [46], in which they were successfully used to treat a spinal infection caused by pandrug-resistant Pseudomonas aeruginosa. These phages were confirmed to specifically infect and lyse the patient’s Pseudomonas aeruginosa strain, including its persistent small-colony variant. The genomes of these phages are highly similar, with vB_PaeP_4032 and vB_PaeP_4034 differing by only five single-nucleotide polymorphisms. All three belong to the genus Limuavirus, which offers a meaningful challenge for models to distinguish subtle sequence variations and identify specific genomic patterns.

We employ PhageCGRNet to perform host prediction based on the genomic sequences of these three phages and compare the results with their known hosts. The results are summarized in Table 9. The results demonstrate that PhageCGRNet correctly predicted Pseudomonas aeruginosa as the host for all three phages, with prediction probability exceeding 0.9999. This demonstrates the model’s high-confidence and accurate prediction capability in this case. In comparison, RaFAH [11], vHULK [12], CL4PHI [25], and PHPGCA [24] also correctly predicted the host, although with lower prediction probability than PhageCGRNet. However, DeepHos t[22] misidentified phage vB_PaeP_4032 as Gordonia terrae, CHERRY [23] erroneously predicted phage vB_PaeP_4032 as Pseudomonas syringae, and PB-LKS [27] incorrectly assigned phage vB_PaeP_4034 to Synechococcus sp. WH 7805. Notably, the values reported for CL4PHI [25] are distances; all other methods provide probability values.

**Table 9:** PhageCGRNet predictions for three phage genomes.

Method	phage vB_PaeP_4029	phage vB_PaeP_4032	phage vB_PaeP_4034
RaFAH [11]	Pseudomonas aeruginosa(0.995600)	Pseudomonas aeruginosa(0.9785)	Pseudomonas aeruginosa(0.965887)
vHULK [12]	Pseudomonas aeruginosa(0.990002)	Pseudomonas aeruginosa(0.98)	Pseudomonas aeruginosa(0.995815)
DeepHost [22]	Pseudomonas aeruginosa(0.997782)	Gordonia terrae(0.520924)	Pseudomonas aeruginosa(0.970926)
CHERRY [23]	Pseudomonas aeruginosa(0.897841)	Pseudomonas syringae (0.612890)	Pseudomonas aeruginosa(0.960873)
PHPGCA [24]	Pseudomonas aeruginosa(0.907802)	Pseudomonas aeruginosa(0.870325)	Pseudomonas aeruginosa(0.933319)
CL4PHI [25]	Pseudomonas aeruginosa(1.095909)	Pseudomonas aeruginosa(0.799409)	Pseudomonas aeruginosa(0.824700)
PB-LKS [27]	Pseudomonas aeruginosa(0.736600)	Pseudomonas aeruginosa(0.711400)	Synechococcus sp. WH 7805(0.757200)
PhageCGRNet	Pseudomonas aeruginosa(0.999998)	Pseudomonas aeruginosa(0.999917)	Pseudomonas aeruginosa(0.999918)

Furthermore, we analyzed the feature contributions of the three phages. By calculating SHAP values, we identified that the sequence pattern with the highest contribution to the prediction outcome corresponds to the *k*-mer GTGCAGG. Further analysis revealed that GTGCAGG repeatedly occurs within the gene region encoding a putative tail fiber protein. The accurate host classification achieved by our model stems precisely from its integration of *k*-mer frequency with CGR positional information.

## 3. Discussion and conclusion

In this study, we proposed a novel deep learning method named PhageCGRNet for the prediction of phage host classification. Due to the variable length of phage genome sequences, our method represents these sequences as three-dimensional matrices of uniform size. The three-dimensional matrix contains two types of information: the frequency information of the *k*-mers obtained from the sequence and the position information of the *k*-mers obtained from the CGR plots. First, we extracted the *k*-mers frequency information of the phage genome sequences. Next, we map the sequences onto CGR plots. For each *k*-mer, we find the average position of the subsequence ending with it in the CGR plot, which serves as the position information for that *k*-mer. In this way, we obtained a new measure of *k*-mers such that the sequence can be represented by this new measure of *k*-mers. Finally, we used the 3D feature matrices as inputs to the CNN model, which can handle multiple channels of inputs. The CNN employs a convolutional kernel to scan and sum the data from each channel individually, thereby combining the information from all channels for a final prediction.

The experimental results demonstrate that our method exhibits superior performance compared with other state-of-the-art methods on the two datasets. Meanwhile, we found in our ablation experiments that the position information by CGR contributes more accuracy than the frequency information. The efficacy of CGR-derived positional information can be understood in the context of its proven ability to resolve biologically significant sequence patterns. Furthermore, CGR can identify biologically relevant sequence patterns. For example, Deschavanne et al. [39] observed through CGR images that four seven-letter words oligonucleotides (GGCGATC, GCGATCG, CGATCGC, and GATCGCC) exhibit exceptionally high frequencies in Synechocystis sp.. Robinson et al. [47] further confirmed that the HIP1 (5’-GCGATCGC-3’) is highly enriched in cyanobacterlum Synechococcus. This octamer may indirectly regulate metallothionein expression related to environmental stress responses by modulating mRNA stability or translation termination efficiency. Additionally, Scholz et al. [48] identified Cas6 as HIP1 in the Synechocystis sp. (Syn6803), demonstrating its ability to recognize and cleave palindromic repeat structures in pre-crRNA to generate mature crRNA. This process is a critical step in the CRISPR-Cas system, as crRNA guides effector proteins like Cas9 to cleave target DNA sequences (e.g., those from invading phages or plasmids). This case not only demonstrates ability of CGR to capture functional sequence patterns but also suggests that these patterns may hold biological significance. Our ablation experiments show that combining the two features achieves a higher classification accuracy than using either feature type alone. We employed the combination of both features to obtain better accuracy than other advanced methods. Indeed, capturing sequence similarity is key to phage–host prediction. Our combined *k*-mer frequency and position features not only enhance similarity representation but also encode genomic patterns with potential biological relevance, as supported by functional enrichment in host-interaction genes (Section 2.8). In future work, we plan to explore more advanced deep learning architectures such as Transformer-based models and Graph Neural Networks (GNNs) to better capture long-range dependencies and structural relationships within phage genomes. Additionally, we will investigate hybrid models that integrate multiple single models (e.g., combining CNNs with recurrent or attention mechanisms) to enhance feature representation and generalization. Interpretability of predictions will also be a focus, using techniques like attention visualization to identify biologically meaningful *k*-mer patterns. Finally, we aim to extend our method to predict broader host ranges and integrate multimodal data (e.g., protein sequences or protein structure) to further improve classification accuracy and practical utility.

## 4. Methods

**Fig 8:**
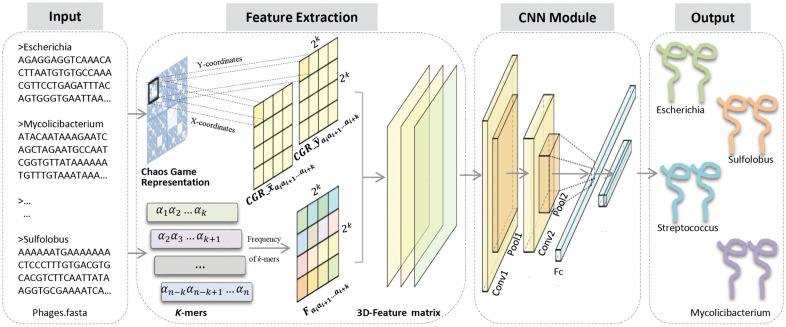
Overall process illustration of our proposed PhageCGRNet. First, the genome sequence of each phage is represented as a three-dimensional matrix, which is then fed into the CNN for the purpose of host prediction.

Our proposed method comprises four core components: (i) input, (ii) feature extraction, (iii) CNN module, and (iv) output, as illustrated in Fig 8. In the input stage, we input the genomic sequences of phages. These sequences are composed of four nucleotide bases, denoted by the letters A, T, G, and C. Since the lengths of these sequences vary, it is essential to extract features from them. Feature extraction helps to represent the sequences and achieves uniformity in the size of feature matrices or feature vectors. Here, we integrate the frequency information of *k*-mers obtained from sequences with the position information of *k*-mers obtained from CGR plots. This process constitutes our feature extraction stage. The integrated feature matrix is then fed into the CNN model in order to achieve the task of classifying the hosts of phages. Finally, the model outputs the classification predictions. We also offer a detailed breakdown of each part in the subsequent sections. Section 4.1 covers the input processing and feature extraction, Section 4.2 details the CNN model, and Section 4.3 describes the model training and output results.

### 4.1. Generation of the feature matrix

**Fig 9:**
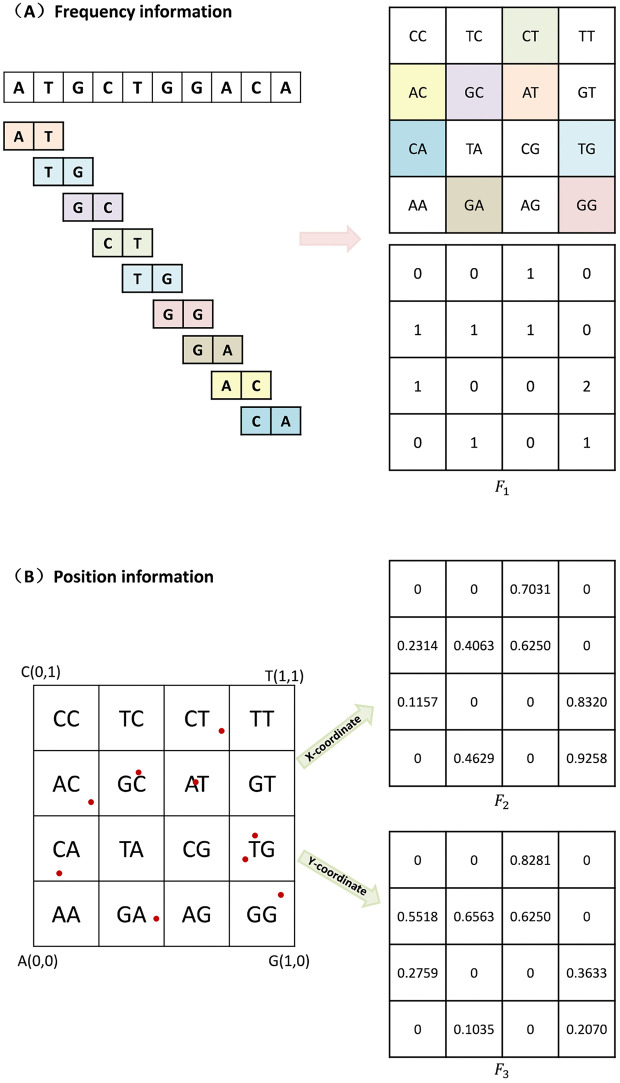
The process of feature matrix construction. (A) shows the DNA sequence being decomposed into *k*-mers (*k* = 2) to generate a frequency matrix (${𝐅}_{\text{1}}$). (B) shows the sequence being projected onto a CGR plot (the red dots represent the position of each nucleotide in the CGR) to derive two positional matrices (*F₂* and *F₃* for the x and y coordinates, respectively).


First, we input the phage genome sequences. Given that ${S=a}_{\text{1}}a_{\text{2}}\ldots a_{L}$ is a DNA sequence of length *L*, where for any i∈{1,2,…,L}, $a_{i}\in \{A,C,G,T\}$, $a_{\text{1}}a_{\text{2}}\ldots a_{k}\;$ is a *k*-mer. First, we count the times all *k*-mer occurrences on this sequence, denoted as $N_{a_{\text{1}}a_{\text{2}}\ldots a_{k}}$. Then, based on the arrangement of CGR illustrated in Fig 9(A), we record the frequencies of *k*-mers in a ${\text{2}}^{k}\times {\text{2}}^{k}$ matrix. This matrix is denoted as the feature matrix $F_{\text{1}}$, which is formulated as follows:

$$\begin{array}{c} F_{\text{1}}=\left[(\begin{array}{ccc} N_{\text{1},\text{1}} & \cdots & N_{{\text{1},\text{2}}^{k}}\\ \vdots & \ddots & \vdots \\ N_{{\text{2}}^{k},\text{1}} & \cdots & N_{{\text{2}}^{k}{,\text{2}}^{k}} \end{array})\right]. \end{array}$$ (5)

Next, each nucleotide in the DNA sequence is mapped one-to-one onto the unit plane using the following iterative function system (Equation (6)), thereby generating a CGR plot.


$$\;{CGR}_{i}=\text{0}.\text{5}\left({CGR}_{i-\text{1}}+\alpha_{i}\right),$$ (6)

where $\;{CGR}_{\text{0}}=(\text{0}.\text{5},\text{0}.\text{5})$, $\alpha_{i}=\left\{ \begin{array}{c} @c(\text{0},\text{0}),if\;\alpha_{i}\;is\;A\\ (\text{0},\text{1}),if\;\alpha_{i}\;is\;C\\ (\text{1},\text{0}),if\;\alpha_{i}\;is\;G\\ (\text{1},\text{1}),if\;\alpha_{i}\;is\;T \end{array}\right.\;\;\;,\;i\in \;\{\text{1},\text{2},\ldots ,\text{L}\}.$

We know that for each *k*-mer in a DNA sequence, we can identify all the subsequence that end with this particular *k*-mer. Subsequently, we can obtain the CGR for each of these subsequences. Specifically, we denote the position of the subsequence ending with a given *k*-mer $a_{\text{1}}a_{\text{2}}\ldots a_{k}$ in the CGR plot as (${CGR\_x_{i}}_{a_{\text{1}}a_{\text{2}}\ldots a_{k}},\;{CGR\_y_{i}}_{a_{\text{1}}a_{\text{2}}\ldots a_{k}}$). It is important to note that in a sequence, a *k*-mer may appear multiple times. As a result, the subsequence ending with that *k*-mer has multiple corresponding coordinates in the CGR plot. To handle this situation, we calculate the average positions ${CGR\_\overset{―}{x}}_{a_{\text{1}}a_{\text{2}}\ldots a_{k}}$ and ${CGR\_\overset{―}{y}}_{a_{\text{1}}a_{\text{2}}\ldots a_{k}}$ for those multiple coordinates [49].


$$\;{CGR\_\overset{―}{x}}_{a_{\text{1}}a_{\text{2}}\ldots a_{k}}=\frac{\left({CGR_{x_{\text{1}}}}_{a_{\text{1}}a_{\text{2}}\ldots a_{k}}+\;{CGR_{x_{\text{2}}}}_{a_{\text{1}}a_{\text{2}}\ldots a_{k}}\cdots \;+{CGR_{x_{N_{A}}}}_{a_{\text{1}}a_{\text{2}}\ldots a_{k}}\right)}{N_{A}},$$ (7)

$$\;{CGR\_\overset{―}{y}}_{a_{\text{1}}a_{\text{2}}\ldots a_{k}}=\frac{\left({CGR_{y_{\text{1}}}}_{a_{\text{1}}a_{\text{2}}\ldots a_{k}}+\;{CGR_{y_{\text{2}}}}_{a_{\text{1}}a_{\text{2}}\ldots a_{k}}\cdots \;+{CGR_{y_{N_{A}}}}_{a_{\text{1}}a_{\text{2}}\ldots a_{k}}\right)}{N_{A}},$$ (8)

where $N_{A}=$ $N_{a_{\text{1}}a_{\text{2}}\ldots a_{k}}$ is the number of times the *k*-mer $a_{\text{1}}a_{\text{2}}\ldots a_{k}$ appears in this sequence.

Afterwards, we obtain matrices $F_{\text{2}}$ and $F_{\text{3}}$ by considering ${CGR\_\overset{―}{x}}_{a_{\text{1}}a_{\text{2}}\ldots a_{k}}$ and ${CGR\_\overset{―}{y}}_{a_{\text{1}}a_{\text{2}}\ldots a_{k}}$ as the second and third features respectively. This is represented as follows:


$$\begin{array}{c} F_{\text{2}}=\left[(\begin{array}{ccc} {CGR\_\overset{―}{x}}_{\text{1},\text{1}} & \cdots & {CGR\_\overset{―}{x}}_{{\text{1},\text{2}}^{k}}\\ \vdots & \ddots & \vdots \\ {CGR\_\overset{―}{x}}_{{\text{2}}^{k},\text{1}} & \cdots & {CGR\_\overset{―}{x}}_{{\text{2}}^{k}{,\text{2}}^{k}} \end{array})\right]\;, \end{array}$$ (9)

$$\begin{array}{c} F_{\text{3}}=\left[(\begin{array}{ccc} {CGR\_\overset{―}{y}}_{\text{1},\text{1}} & \cdots & {CGR\_\overset{―}{y}}_{{\text{1},\text{2}}^{k}}\\ \vdots & \ddots & \vdots \\ {CGR\_\overset{―}{y}}_{{\text{2}}^{k},\text{1}} & \cdots & {CGR\_\overset{―}{y}}_{{\text{2}}^{k}{,\text{2}}^{k}} \end{array})\right]. \end{array}$$ (10)

To illustrate how to generate the 3D matrix, we present an example in Fig 9. Assume that we have sequence S = AGTCGTTACA and the length of the *k*-mer is set to 2. Fig 9(A) demonstrates the process of calculating the frequencies of *k*-mers. We represent the frequency information as a matrix, where the element coordinates are arranged according to the CGR. In Fig 9(B), we can see the process of mapping the DNA sequences onto the CGR plot. After the mapping, we calculate the mean position of the corresponding *k*-mers in the CGR plot, which results in the values of $F_{\text{2}}$ and $F_{\text{3}}$, respectively.

Since that Matrix $F_{\text{1}}$ denotes the frequency of *k*-mers, while matrices $F_{\text{2}}$ and $F_{\text{3}}$ are obtained from the coordinates of the point positions in the CGR plot, there is a need to unify the measures scales. To achieve this, we normalize these three matrices. The normalization process is presented as follows:


$$X_{normalize}=\frac{x_{i}-x_{min}}{x_{max}-x_{min}}.$$ (11)

Additionally, we provide the detailed computational process for matrices $F_{\text{1}}$, $F_{\text{2}}$ and $F_{\text{3}}$. If sequence *S* = AGTCGTTACA, *k* = 2.


$$N_{AA}=\text{0},\;N_{AC}=\text{1},\;N_{AG}=\text{1},\;N_{AT}=\text{0},\;N_{CA}=\text{1},\;N_{CC}=\text{0},\;N_{CG}=\text{1},\;N_{CT}=\text{0},$$

$$N_{GA}=\text{0},\;N_{GC}=\text{0},\;N_{GG}=\text{0},\;N_{GT}=\text{2},\;N_{TA}=\text{1},\;N_{TC}=\text{1},\;N_{TG}=\text{0},N_{TT}=\text{1}.$$

$$\begin{array}{c} F_{\text{1}}=(\begin{array}{ccc} N_{CC} & N_{GC} & \begin{array}{cc} N_{CG} & N_{GG} \end{array}\\ N_{AC} & N_{TC} & \begin{array}{cc} N_{AG} & N_{TG} \end{array}\\ \begin{array}{c} N_{CA}\\ N_{AA} \end{array} & \begin{array}{c} N_{GA}\\ N_{TA} \end{array} & \begin{array}{cc} \begin{array}{c} N_{CT}\\ N_{AT} \end{array} & \begin{array}{c} N_{GT}\\ N_{TT} \end{array} \end{array} \end{array})=(\begin{array}{ccc} \text{0} & \text{0} & \begin{array}{cc} \text{1} & \text{0} \end{array}\\ \text{1} & \text{1} & \begin{array}{cc} \text{1} & \text{0} \end{array}\\ \begin{array}{c} \text{1}\\ \text{0} \end{array} & \begin{array}{c} \text{0}\\ \text{1} \end{array} & \begin{array}{cc} \begin{array}{c} \text{0}\\ \text{0} \end{array} & \begin{array}{c} \text{2}\\ \text{1} \end{array} \end{array} \end{array}) \end{array}$$

The positional coordinates of sequence S on the CGR are as follows: A: (0.25, 0.25),

AG: (0.625, 0.625), ${CGR}_{AG}=(\text{0}.\text{6}\text{2}\text{5},\;\text{0}.\text{6}\text{2}\text{5})$;

AGT: (0.8125, 0.3125), ${CGR}_{GT}=(\text{0}.\text{8}\text{1}\text{2}\text{5},\;\text{0}.\text{3}\text{1}\text{2}\text{5})$;

AGTC: (0.40625, 0.65625), ${CGR}_{TC}=(\text{0}.\text{4}\text{0}\text{6}\text{2}\text{5},\;\text{0}.\text{6}\text{5}\text{6}\text{2}\text{5})$;

AGTCG: (0.703125, 0.828125), ${CGR}_{CG}=(\text{0}.\text{7}\text{0}\text{3}\text{1}\text{2}\text{5},\;\text{0}.\text{8}\text{2}\text{8}\text{1}\text{2}\text{5})$;

AGTCGT: (0.8515625, 0.4140625), ${CGR}_{GT}=(\text{0}.\text{8}\text{5}\text{1}\text{5}\text{6}\text{2}\text{5},\;\text{0}.\text{4}\text{1}\text{4}\text{0}\text{6}\text{2}\text{5})$;

AGTCGTT: (0.92578125, 0.202703125), ${CGR}_{TT}=(\text{0}.\text{9}\text{2}\text{5}\text{7}\text{8}\text{1}\text{2}\text{5},\;\text{0}.\text{2}\text{0}\text{7}\text{0}\text{3}\text{1}\text{2}\text{5})$;

AGTCGTTA: (0.462890625, 0.103515625), ${CGR}_{TA}=(\text{0}.\text{4}\text{6}\text{2}\text{8}\text{9}\text{0}\text{6}\text{2}\text{5},\;\text{0}.\text{1}\text{0}\text{3}\text{5}\text{1}\text{5}\text{6}\text{2}\text{5})$;

AGTCGTTAC: (0.2314453125, 0.5517578125),


$${CGR}_{AC}=(\text{0}.\text{2}\text{3}\text{1}\text{4}\text{4}\text{5}\text{3}\text{1}\text{2}\text{5},\;\text{0}.\text{5}\text{5}\text{1}\text{7}\text{5}\text{7}\text{8}\text{1}\text{2}\text{5});\;$$

AGTCGTTACA: (0.11572265625, 0.27587890625),


$${CGR}_{CA}=(\text{0}.\text{1}\text{1}\text{5}\text{7}\text{2}\text{2}\text{6}\text{5}\text{6}\text{2}\text{5},\;\text{0}.\text{2}\text{7}\text{5}\text{8}\text{7}\text{8}\text{9}\text{0}\text{6}\text{2}\text{5}).$$

Therefore, the average positions: ${\text{CGR}\_\overset{―}{\text{x}}}_{\text{AA}}=\text{0},\;{\text{CGR}\_\overset{―}{\text{y}}}_{\text{AA}}=\text{0}$;


$${\text{CGR}\_\overset{―}{\text{x}}}_{\text{AC}}=\text{0}.\text{2314453125},\;{\text{CGR}\_\overset{―}{\text{y}}}_{\text{AC}}=\text{0}.\text{5517578125};\;$$

$${\text{CGR}\_\overset{―}{\text{x}}}_{\text{AG}}=\text{0}.\text{625},\;{\text{CGR}\_\overset{―}{\text{y}}}_{\text{AG}}=\text{0}.\text{625};\;$$

$${\text{CGR}\_\overset{―}{\text{x}}}_{\text{AT}}=\text{0},\;{\text{CGR}\_\overset{―}{\text{y}}}_{\text{AT}}=\text{0};\;$$

$${\text{CGR}\_\overset{―}{\text{x}}}_{\text{CA}}=\text{0}.\text{11572265625},\;{\text{CGR}\_\overset{―}{\text{y}}}_{\text{CA}}=\text{0}.\text{27587890625};\;$$

$${\text{CGR}\_\overset{―}{\text{x}}}_{\text{CC}}=\text{0},\;{\text{CGR}\_\overset{―}{\text{y}}}_{\text{CC}}=\text{0};\;$$

$${\text{CGR}\_\overset{―}{\text{x}}}_{\text{CG}}=\text{0}.\text{703125},\;{\text{CGR}\_\overset{―}{\text{y}}}_{\text{CG}}=\text{0}.\text{828125};\;$$

$${\text{CGR}\_\overset{―}{\text{x}}}_{\text{CT}}=\text{0},\;{\text{CGR}\_\overset{―}{\text{y}}}_{\text{CT}}=\text{0};\;$$

$${\text{CGR}\_\overset{―}{\text{x}}}_{\text{GA}}=\text{0},\;{\text{CGR}\_\overset{―}{\text{y}}}_{\text{GA}}=\text{0};\;$$

$${\text{CGR}\_\overset{―}{\text{x}}}_{\text{GC}}=\text{0},\;{\text{CGR}\_\overset{―}{\text{y}}}_{\text{GC}}=\text{0};\;$$

$${\text{CGR}\_\overset{―}{\text{x}}}_{\text{GG}}=\text{0},\;{\text{CGR}\_\overset{―}{\text{y}}}_{\text{GG}}=\text{0};\;$$

$${\text{CGR}\_\overset{―}{\text{x}}}_{\text{GT}}=\frac{\text{0}.\text{8125}+\text{0}.\text{8515625}}{\text{2}}=\text{0}.\text{83203125},\;{\text{CGR}\_\overset{―}{\text{y}}}_{\text{GT}}=\frac{\text{0}.\text{3125}+\text{0}.\text{4140625}}{\text{2}}=\text{0}.\text{36328125};\;$$

$${\text{CGR}\_\overset{―}{\text{x}}}_{\text{TA}}=\text{0}.\text{462890625},\;{\text{CGR}\_\overset{―}{\text{y}}}_{\text{TA}}=\text{0}.\text{103515625};$$

$${\text{CGR}\_\overset{―}{\text{x}}}_{\text{TC}}=\text{0}.\text{40625},\;{\text{CGR}\_\overset{―}{\text{y}}}_{\text{TC}}=\text{0}.\text{65625};\;$$

$${\text{CGR}\_\overset{―}{\text{x}}}_{\text{TG}}=\text{0},\;{\text{CGR}\_\overset{―}{\text{y}}}_{\text{TG}}=\text{0};\;$$

$${\text{CGR}\_\overset{―}{\text{x}}}_{\text{TT}}=\text{0}.\text{92578125},\;{\text{CGR}\_\overset{―}{\text{y}}}_{\text{TT}}=\text{0}.\text{20703125};\;$$

Hence $F_{\text{2}}$ and $F_{\text{3}}\;$ can be calculated as:


$$\begin{array}{c} F_{\text{2}}=(\begin{array}{ccc} {CGR\_\overset{―}{\text{x}}}_{CC} & {CGR\_\overset{―}{\text{x}}}_{GC} & \begin{array}{cc} {CGR\_\overset{―}{\text{x}}}_{CG} & {CGR\_\overset{―}{\text{x}}}_{GG} \end{array}\\ {CGR\_\overset{―}{\text{x}}}_{AC} & {CGR\_\overset{―}{\text{x}}}_{TC} & \begin{array}{cc} {CGR\_\overset{―}{\text{x}}}_{AG} & {CGR\_\overset{―}{\text{x}}}_{TG} \end{array}\\ \begin{array}{c} {CGR\_\overset{―}{\text{x}}}_{CA}\\ {CGR\_\overset{―}{\text{x}}}_{AA} \end{array} & \begin{array}{c} {CGR\_\overset{―}{\text{x}}}_{GA}\\ {CGR\_\overset{―}{\text{x}}}_{TA} \end{array} & \begin{array}{cc} \begin{array}{c} {CGR\_\overset{―}{\text{x}}}_{CT}\\ {CGR\_\overset{―}{\text{x}}}_{AT} \end{array} & \begin{array}{c} {CGR\_\overset{―}{\text{x}}}_{GT}\\ {CGR\_\overset{―}{\text{x}}}_{TT} \end{array} \end{array} \end{array}) \end{array}$$

$$\begin{array}{c} =(\begin{array}{ccc} \text{0} & \text{0} & \begin{array}{cc} \text{0}.\text{703125} & \text{0} \end{array}\\ \text{0}.\text{2314453125} & \text{0}.\text{40625} & \begin{array}{cc} \text{0}.\text{625} & \text{0} \end{array}\\ \begin{array}{c} \text{0}.\text{11572265625}\\ \text{0} \end{array} & \begin{array}{c} \text{0}\\ \text{0}.\text{462890625} \end{array} & \begin{array}{cc} \begin{array}{c} \text{0}\\ \text{0} \end{array} & \begin{array}{c} \text{0}.\text{83203125}\\ \text{0}.\text{92578125} \end{array} \end{array} \end{array}) \end{array}$$

$$\begin{array}{c} F_{\text{3}}=(\begin{array}{ccc} {CGR\_\overset{―}{\text{y}}}_{CC} & {CGR\_\overset{―}{\text{y}}}_{GC} & \begin{array}{cc} {CGR\_\overset{―}{\text{y}}}_{CG} & {CGR\_\overset{―}{\text{y}}}_{GG} \end{array}\\ {CGR\_\overset{―}{\text{y}}}_{AC} & {CGR\_\overset{―}{\text{y}}}_{TC} & \begin{array}{cc} {CGR\_\overset{―}{\text{y}}}_{AG} & {CGR\_\overset{―}{\text{y}}}_{TG} \end{array}\\ \begin{array}{c} {CGR\_\overset{―}{\text{y}}}_{CA}\\ {CGR\_\overset{―}{\text{y}}}_{AA} \end{array} & \begin{array}{c} {CGR\_\overset{―}{\text{y}}}_{GA}\\ {CGR\_\overset{―}{\text{y}}}_{TA} \end{array} & \begin{array}{cc} \begin{array}{c} {CGR\_\overset{―}{\text{y}}}_{CT}\\ {CGR\_\overset{―}{\text{y}}}_{AT} \end{array} & \begin{array}{c} {CGR\_\overset{―}{\text{y}}}_{GT}\\ {CGR\_\overset{―}{\text{y}}}_{TT} \end{array} \end{array} \end{array}) \end{array}$$

$$\begin{array}{c} =(\begin{array}{ccc} \text{0} & \text{0} & \begin{array}{cc} \text{0}.\text{828125} & \text{0} \end{array}\\ \text{0}.\text{5517578125} & \text{0}.\text{65625} & \begin{array}{cc} \text{0}.\text{625} & \text{0} \end{array}\\ \begin{array}{c} \text{0}.\text{27587890625}\\ \text{0} \end{array} & \begin{array}{c} \text{0}\\ \text{0}.\text{103515625} \end{array} & \begin{array}{cc} \begin{array}{c} \text{0}\\ \text{0} \end{array} & \begin{array}{c} \text{0}.\text{36328125}\\ \text{0}.\text{20703125} \end{array} \end{array} \end{array}) \end{array}$$

### 4.2. Convolutional neural network

Following the construction of three-dimensional matrices representation of the phage sequences, we utilize a CNN model to further enhance the features and to classify the hosts. Specifically, we input the 3D matrices into the CNN after normalization. We design the CNN architecture with two convolutional layers. Each convolutional layer is followed by a pooling layer, where we adopt the MaxPool method [50]. In addition, The rectified linear unit (ReLU) function [51], defined as *f(x)* = max{0, *x*}, is applied to enhance the nonlinear expression ability of the model at each layer. The following stage is the flattening of these features, which is carried out in order to facilitate their conversion into one-dimensional vectors. After that, we introduce a fully connected layer. This layer connects every neuron from the previous layer to all neurons in the current layer, enabling the model to learn complex relationships among the features. Finally, the output of the fully connected layer is fed into the prediction layer. Here, we use the Softmax activation function [52] to calculate the score for each host taxonomy. The Softmax function serves to convert the raw output values into a probability distribution. The classification with the highest probability score is then selected as the model’s prediction.

### 4.3. Model training

In the training process, we employ the cross entropy loss function [53] to quantify the discrepancy between the predicted probability distribution and the true labels of the host classifications. The cross entropy loss is a widely used metric in classification tasks. For multi-class classification, we use the categorical cross-entropy loss, given by:


$$L\left(y,\hat{y}\right)=-\sum_{\partial =\text{1}}^{N}y_{\partial }\text{log}\left({\hat{y}}_{\partial }\right),$$ (12)

where, *N* is the number of classes, $y_{\partial }$ is the true label for class ∂, ${\hat{y}}_{\partial }\;$is the predicted probability for class ∂. To optimize the parameters of our CNN model, we utilize the Adam optimization algorithm [54]. We set the learning rate of this algorithm to 0.001*.* Finally, the model outputs the classification prediction results, predicting the category of each host based on the learnt features.

### 4.4. Distance calculation

We transpose the 3D matrix composed of the *k*-mer frequency information and the CGR position information into a vector. The elements of this vector are arranged in a specific order, namely the *k*-mer frequencies, the average value of the *x*-coordinates of CGR, and the average value of the *y*-coordinates of CGR. After the vector transformation is completed, we compute sequence similarity using Equation (13) and subsequently derived sequence distances via Equation (14). Finally, the Neighbor-Joining algorithm [45] is employed to construct the phylogenetic tree.


$${similarity}_{cos}=\frac{{Vector}_{\text{1}}\cdot {Vector}_{\text{2}}}{{\left\|{Vector}_{\text{1}}\right\|}_{\text{2}}\cdot {\left\|{Vector}_{\text{2}}\right\|}_{\text{2}}}\;$$ (13)

$${distance}_{cos}=\text{1}-{similarity}_{cos}$$ (14)
